# Enhancement of Structural Stability and IgG Affinity of a *Z34C*-Derived α-Helical Peptide via Lactam Stapling

**DOI:** 10.3390/antib14040108

**Published:** 2025-12-16

**Authors:** Jung Gu Lee, Inseo Lee, Joo-young Kim, Suin Kim, Woo-jin Jeong, Ji-eun Kim

**Affiliations:** 1 Department of Biological Sciences and Bioengineering, Inha University, Incheon 22212, Republic of Korea; dlwjdrn9961@inha.edu (J.G.L.); islee@inha.edu (I.L.); jyk3927@inha.edu (J.-y.K.); db_suinkim@inha.edu (S.K.); 2 Department of Biological Engineering, Inha University, Incheon 22212, Republic of Korea; 3 Department of Chemical and Biochemical Engineering, Dongguk University, Seoul 22012, Republic of Korea

**Keywords:** lactam stapling, Fc-binding peptide, α-helical peptide, Z34C domain

## Abstract

Background: The Fc region of immunoglobulin G (IgG) is a key target in therapeutic and analytical applications, such as antibody purification and site-specific bioconjugation. Although Protein A exhibits strong Fc-binding affinity, its large molecular weight and limited chemical flexibility pose challenges for use in compact or chemically defined systems. To address these limitations, we designed two α-helical peptides, SpA h1 and SpA h2, based on the Fc-binding helices of the Z34C domain from *Staphylococcus aureus* Protein A. Method: To enhance the structural stability and Fc-binding capability of these peptides, a lactam-based stapling strategy was employed by introducing lysine and glutamic acid residues at positions *i* and *i* + 4. Result: The resulting stapled peptides, (s)SpA h1 and (s)SpA h2, exhibited significantly improved α-helical content and IgG-binding performance, as demonstrated by circular dichroism (CD) spectroscopy and fluorescence-based IgG capture assays. Surface plasmon resonance (SPR) analysis confirmed specific, concentration-dependent interactions with the Fc region of human IgG, with (s)SpA h1 consistently showing the binding affinity and stability. Proteolytic resistance assays using α-chymotrypsin revealed that (s)SpA h1 maintained its structural integrity over time, exhibiting markedly enhanced resistance to enzymatic degradation compared to its linear counterpart. Furthermore, (s)SpA h1 exhibited strong Fc selectivity with minimal Fab affinity, confirming its suitability as a compact and Fc-specific binding ligand. Conclusions: These results confirm the successful design and development of structurally reinforced Fc-binding peptides that overcome the inherent limitations of short linear sequences through both high-affinity sequence optimization and lactam-based stapling. Among them, (s)SpA h1 demonstrates the most promising characteristics as a compact yet stable Fc-binding ligand, suitable for applications such as antibody purification and site-specific bioconjugation.

## 1. Introduction

Immunoglobulin G (IgG), with high specificity and affinity, is a major biopharmaceutical modality widely applied in immune recognition, therapeutic targeting, antibody purification, and diverse biotechnological applications [1,2]. In particular, the Fc region of IgG has been thoroughly investigated as a binding target for protein- or peptide-based ligands, thereby enabling applications in antibody purification, drug delivery, such as Fc fusion proteins [3,4,5], IgG-nanoparticle system [6], antibody-drug conjugates, and diagnostic platforms [7,8,9,10,11]. Moreover, IgG Fc affinity ligands have garnered increasing attention in antibody-mediated drug delivery owing to their biocompatibility, high-affinity and selective binding to antibodies or Fc-fusion proteins, and the stability of noncovalent affinity complexes under physiologically relevant conditions [12]. Such IgG Fc affinity ligands demonstrate outstanding efficiency in antibody purification and drug delivery, owing to their exceptional selectivity and specificity toward IgG [13]. Among the various Fc-binding ligands, Protein A (a virulence factor produced by *Staphylococcus aureus*) has been used in the chromatographic purification of Abs due to its high affinity and specificity towards IgGs from different species [14,15,16]. However, despite its key advantageous features, Protein A-based purification systems face several limitations, including their large molecular size, limited chemical flexibility, high resin cost, harsh conditions, and reduced operational lifespan. Moreover, the clinical applicability of Staphylococcal Protein A (SpA) is inherently limited by its bacterial origin and the associated risk of immunogenic responses in humans, particularly under conditions involving high dosages or repeated administrations. Accordingly, there is a substantial demand for alternative ligands capable of affinity capture of IgG with enhanced safety and performance profiles. In this context, to overcome the limitations associated with Protein A, Fc-binding peptides have emerged as promising alternatives [17]. These peptides present several advantages, including small molecular size, ease of synthesis, cost-effective large-scale production with consistent quality, mild elution conditions, and tunable binding affinities, making them attractive candidates to replace conventional Protein A as IgG affinity ligands [18,19,20,21]. Furthermore, the application of peptides entails negligible immunogenic risk, which can be further attenuated through advanced engineering strategies, including the incorporation of non-natural amino acids or the modification of the molecular scaffold [22,23,24].

**Figure 1:**
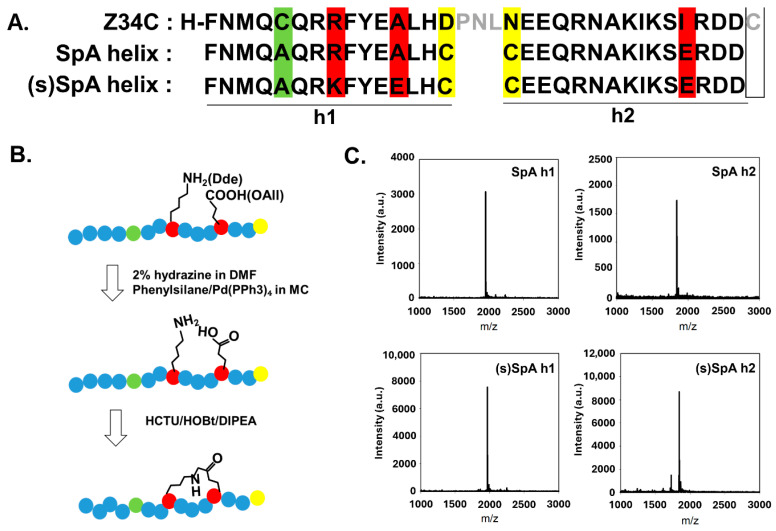
(**A**) Alignment of α-helical Fc-binding peptides derived from Z34C and their stapled analogs. Residues substituted for lactam stapling are shown in red. Yellow highlights indicate cysteine residues introduced for site-specific conjugation. Green highlights mark a cysteine residue in helix 1 replaced with alanine, which does not interfere with Fc binding but may affect conjugation efficiency. Grey indicates amino acids in Z34C that do not correspond to helix 1 or helix 2. (**B**) Schematic of the solid-phase lactam stapling reaction. Stapled peptides were synthesized by Fmoc-SPPS using Lys(Dde) and Glu(OAll). (**C**) MALDI-TOF MS spectra of SpA h1(calculated MW: 1954.89, observed MW: 1956.06), SpA h2 (calculated MW: 1845.9, observed MW: 1846.84), (s)SpA h1(calculated MW: 1968.23, observed MW: 1967.75), (s)SpA h2 (calculated MW: 1844.97, observed MW: 1845.06).

In this study, we designed and synthesized peptides based on the sequences of Helix 1 (SpA h1) and Helix 2 (SpA h2) within the Z34C domain of *Staphylococcus aureus* Protein A, which contains the Fc-binding motif (Figure 1). Single-helix peptides derived from Z34C lack the stabilizing disulfide bond, leading to reduced Fc-binding affinity, as disruption of the α-helical conformation significantly impairs binding performance [25,26]. Therefore, strategies that reinforce the α-helical structure are essential to restore and enhance the Fc-binding capability of these peptides [27,28]. In response, peptide stapling techniques have been developed to stabilize α-helical structures through covalent cross-linking [29,30]. Among these approaches, lactam bridges formed between lysine and glutamic acid residues offer a biocompatible and synthetically accessible method for helix stabilization [31]. Accordingly, a lactam-based stapling strategy was employed to enhance the structural stability of the peptides [32]. This covalent cross-linking reinforces the α-helical conformation of the peptide, rendering it less susceptible to proteolytic cleavage and thereby increasing its half-life and overall stability [33,34]. For instance, early work using short growth hormone-releasing factor (GHRF) analogues demonstrated greater helicity and preserved bioactivity upon lactamisation of a Lys–Asp *i*, *i* + 4 pair [35]. More recently, a cell-penetrant lactam-stapled peptide mimicking the eIF4E-binding motif of 4E-BP1 was designed to disrupt eIF4E–protein interactions, thereby inhibiting cap-dependent translation and suppressing tumorigenic activity [36]. Furthermore, bicyclic stapled peptides based on p53 were developed by combining all-hydrocarbon stapling and lactam stapling strategies, yielding constructs such as p53-16, which exhibited nanomolar affinity toward MDM2 and MDMX and selectively activated the p53 pathway to inhibit tumor cell growth in vitro [37]. This strategy serves as a valuable tool for the development of stable, drug-like peptides with potential therapeutic applications.

Based on prior studies identifying the key amino acid residues in the Z34C sequence involved in Fc binding and their respective contributions [38], residues in the SpA h1 and SpA h2 sequences that do not directly participate in binding with the Fc region were substituted with those required for lactam stapling or site-specific conjugation. This design allowed the resulting helix-based peptides derived from Z34C to retain their ability to bind the Fc region of IgG. We hypothesized that the incorporation of lactam-based stapling would enhance the α-helical content of the peptides, thereby improving their Fc-binding affinity relative to their unstapled counterparts. To validate this hypothesis, we systematically evaluated the synthesized peptides with respect to their secondary structure, Fc-binding affinity, and proteolytic stability.

## 2. Materials and Methods

### 2.1. Peptide Synthesis

Helix and stapled helix peptides were synthesized using standard Fmoc solid-phase peptide synthesis (SPPS) protocols on Rink Amide MBHA resin LL (Novabiochem, Darmstadt, Germany [39,40]). All amino acids were protected with standard side-chain protecting groups. Each coupling reaction was performed with 5 equivalents of Fmoc-protected amino acids, 4.5 equivalents of HCTU and HOBt, and 10 equivalents of DIPEA in DMF for 1.5 h per step. Fmoc deprotection was carried out using 20% piperidine in DMF (*v*/*v*) for 30 min. For stapled peptides, Lys (Dde) and Glu (OAll) residues were incorporated at the desired positions (*i* and *i* + 4). After complete elongation of the linear sequence, the Dde protecting group on lysine was removed using 2% hydrazine in DMF. To remove the Oall protecting group from Glu residues, 15 equivalents of phenylsilane and 0.5 equivalents of Pd(PPh3)_4_ were dissolved in methylene chloride (MC), and the reaction was carried out under light-protected conditions with gentle shaking for 1 h. The lactam bridge was subsequently formed via on-resin cyclization between lysine and glutamic acid side chains using DIPEA activation in DMF. Upon completion of synthesis, peptides were cleaved from the resin and globally deprotected using a cleavage cocktail of TFA/EDT/TIS (95:2.5:2.5) for 3 h. The resulting crude peptides were precipitated with tert-butyl methyl ether (TBME) and centrifuged at 4000 rpm for 5 min. Peptides were purified by reverse-phase HPLC on a C4 semi-preparative column (water/acetonitrile with 0.1% TFA). Molecular weights were confirmed by MALDI-TOF MS using CHCA as the matrix. Peptide concentrations were determined by UV–Vis spectrophotometry in a water/acetonitrile (1:1) mixture, using a molar extinction coefficient of 1280 M^−1^·cm^−1^ at 280 nm for tyrosine-containing sequences, and at 214 nm for sequences lacking aromatic residues (e.g., Helix 2-based peptides).

### 2.2. Circular Dichroism (CD)

Circular dichroism (CD) spectra were obtained using aJ-815 (Jasco, Oklahoma City, OK, USA). The measurements were performed on 40 μM peptide solutions in 150 mM KF. CD spectra were recorded over a wavelength range of 250 to 190 nm using a 1 mm-path length cuvette. Each spectrum represents the average of three scans.

### 2.3. IgG Capture Test

#### 2.3.1. Surface Preparation

Epoxy-functionalized microscope glass slides (Tekdon Incorporated, Myakka City, FL, USA) were mounted in eight-well chambers. For peptide immobilization, NH_2_-PEG_5000_-COOH linkers (Biopharma PEG, Watertown, MA, USA) were applied to each well at a concentration of 1 mg/mL in ddH_2_O and incubated for ~5 h. The slides were then rinsed three times with ddH_2_O. Carboxyl groups of NH_2_-PEG_5000_-COOH linkers were activated for 30 min using 4 µg/mL 1-ethyl-3-(3-dimethylaminopropyl) carbodiimide hydrochloride (EDC; Thermo Fisher Scientific, Waltham, MA, USA) and 6 µg/mL N-hydroxy succinimide (NHS; Thermo Fisher Scientific) in ddH_2_O. Upon activation, the surfaces were washed three times with 1× PBS (Gibco, Grand Island, NY, USA) and incubated for over 5 h with an excess of NH_2_-PEG_1000_-MAL linkers (1 mg/mL in 1× PBS; Biopharma PEG). Following a final rinse with 1× PBS, the surfaces were incubated for ~5 h with peptides (100 nmol/mL in 1× PBS). Note that PEG-functionalized surfaces without peptide were used as a negative control, while protein A-immobilized surfaces served as a positive control. For positive control preparation, protein A (1 µg/mL in 1× PBS; Sigma Aldrich, St. Louis, MO, USA) was directly conjugated to the NH_2_-PEG_5000_-COOH linkers following EDC/NHS activation.

#### 2.3.2. Antibody Capture and Analysis

Alexa Fluor^®^ 555-labeled anti-LAG-3 antibodies (Abcam, Cambridge, UK) were used to evaluate the antibody-binding capacity of each functionalized surface. Specifically, 100 µL of antibody solution (5 µg/mL in 1× PBS) was added to each well of an eight-well chamber slide pre-coated with SpA h1, SpA h2, (s)SpA h1, (s)SpA h2, or control surfaces. Samples were incubated at 25 °C for 3 h to facilitate antibody binding. Following incubation, each well was washed three times with 250 µL of 1× PBS to remove unbound antibodies. The amount of surface-captured antibody was quantified via fluorescence imaging. Imaging parameters, including exposure time, were held constant across all samples, and brightness/contrast adjustments were applied uniformly. Fluorescence intensity was analyzed using Image J software (64-bit Java 8).

### 2.4. Surface Plasmon Resonance (SPR)

Binding affinities were evaluated using surface plasmon resonance (SPR) on the iMSPR mini system (iCLUBIO, Seongnam-si, Republic of Korea). Human IgG (BioCell, Irvine, CA, USA) was immobilized onto an HC1000 chip using amine coupling. Stapled helix peptides were injected starting at 200 μM with four serial two-fold dilutions. Association and dissociation were monitored for 2 min each. All measurements were performed in Dulbecco’s Phosphate-Buffered Saline (DPBS).

### 2.5. Molecular Docking

Structural models used for analysis were obtained from the Protein A/Fc complex (PDB ID: 1L6X) and the Protein A/Fab complex (PDB ID: 1DEE) in the Protein Data Bank. To evaluate the binding affinity between (s)SpA h1 and the Fc fragment, the h1 region of the Z34C domain within the 1L6X structure was modified to the (s)SpA h1 sequence. All structures were prepared in the Molecular Operating Environment (MOE) by adding hydrogen atoms and performing energy minimization using the MMFF94x force field. For docking simulations, the Protein A–Fab interaction interface identified in the 1DEE complex was defined as the target binding site. Ligand placement was carried out using the Triangle Matcher algorithm, followed by pose refinement and scoring with the GBVI/WSA function to estimate the binding free energy of each pose.

### 2.6. Protease Resistance Experiment

To evaluate the protease resistance of the helix peptides, SpA h1 and (s)SpA h1 were tested against α-chymotrypsin digestion. Peptides were prepared at a concentration of 100 μM in DPBS, and α-chymotrypsin was added to achieve a final peptide-to-enzyme mass ratio of 100:1. The digestion was carried out at time points of 0, 1, 5, 15, 30, and 60 min, after which 0.1% TFA was added to terminate the reaction. The resulting samples were analyzed by reversed-phase HPLC (water/acetonitrile with 0.1% TFA) to monitor peptide stability.

## 3. Result and Discussion

We designed two α-helical peptides, helix 1 (FNMQAQRRFYEALHC) and helix 2 (CEEQRNAKIKSIRDD), based on Z34C (an IgG-binding peptide derived from protein A, PDB ID: 1L6X), which contain key residues responsible for binding to the Fc region of IgG (Figure 1A and Figure S1). To enhance the structural stability of these peptides, non-essential residues not directly involved in Fc binding were strategically substituted with lysine and glutamic acid at the *i* and *i* + 4 positions, enabling lactam-based stapling (Figure 1B,C and Figure S2). To enable site-specific conjugation and surface immobilization without interfering with Fc-binding activity, each peptide was engineered to contain a terminal cysteine residue bearing a reactive thiol group introduced by substituting the aspartic acid immediately preceding the N-terminus in helix 1 and the asparagine adjacent to the C-terminus in helix [25]. Due to the antiparallel orientation of the two peptides on the protein surface, the cysteine insertion sites were strategically chosen based on the binding orientation to avoid steric interference and facilitate efficient conjugation. Furthermore, the native cysteine residue within the helix 1 sequence was substituted with alanine to eliminate potential structural or functional interference with the helical conformation and Fc-binding capacity. Alanine was chosen because it is known to have the highest propensity to form α-helices among amino acids, thereby further supporting the structural integrity of the peptide [41].

**Figure 2:**
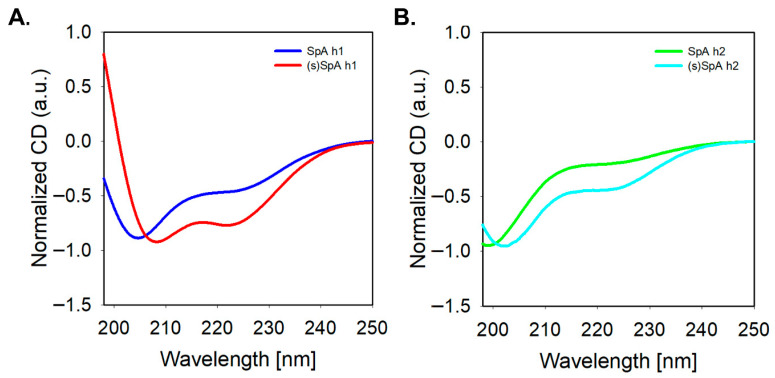
Circular dichroism (CD) analysis of the helix peptides. (**A**) SpA h1 (blue) vs. (s)SpA h1 (red); (**B**) SpA h2 (Green) vs. (s)SpA h2 (Cyan).

To evaluate the secondary structures of the synthesized peptides, circular dichroism (CD) spectroscopy was performed. As shown in Figure 2, both SpA h1 and SpA h2 exhibited characteristic α-helical CD signatures with negative minima at approximately 208 nm and 222 nm, confirming their intrinsic helical propensity in solution. Notably, SpA h1 displayed stronger helicity than SpA h2, as indicated by deeper ellipticity values. Upon lactam stapling, both (s)SpA h1 and (s)SpA h2 showed further stabilization of the helical conformation. Furthermore, the 222/208 ratio of (s)SpA h1 was closer to 1.0 than that of (s)SpA h2, suggesting a more stable and homogeneous α-helical structure [42,43,44]. Taken together, these results indicate that SpA h1 forms a more stable α-helix than SpA h2 even in its linear form, and although lactam stapling enhances helicity in both cases, the overall structural advantage remains more significant for the SpA h1-derived peptide. These findings confirm that the lactam-based stapling strategy effectively reinforces the helical conformation of the peptides and supports the enhanced structural stability of the peptides in aqueous solution.

**Figure 3:**
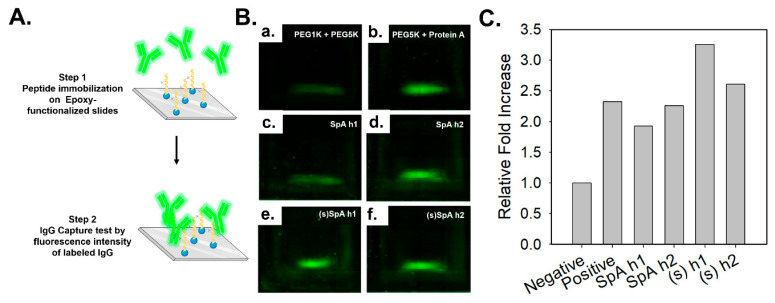
IgG capture assay using FITC-labeled human IgG to evaluate the Fc-binding capability of peptides. (**A**) Design and application of stapled FC binding peptides derived from Protein A (**B**) Fluorescence microscopy images of epoxy-functionalized glass slides treated with: PEG1K + PEG5K as negative control, PEG5K + Protein A as positive control, SpA h1, SpA h2, (s)SpA h1, (s)SpA h2. (**C**) Quantitative fluorescence intensity analysis performed using ImageJ software (64-bit Java 8). (n = 1).

To evaluate the Fc-binding capabilities of the synthesized peptides, an IgG capture assay was conducted using FITC-labeled human IgG. As shown in Figure 3A, each peptide was immobilized onto an epoxy-functionalized glass slide pre-modified with PEG linkers via thiol-maleimide coupling, and FITC-labeled human IgG was applied to assess binding interactions [45]. The negative control showed negligible fluorescence (Figure 3B(a)), while the positive control containing Protein A yielded a strong signal intensity (Figure 3B(b)), validating the assay. The stapled peptides, (s)SpA h1 and (s)SpA h2 (Figure 3B(e,f)), demonstrated increased IgG-binding capacity compared to their unstapled counterparts (Figure 3B(c,d)), suggesting stronger binding to human IgG. Notably, (s)SpA h1 exhibited a significantly higher fluorescence intensity, indicating the highest IgG-binding capacity among the four peptides evaluated. This finding is consistent with the CD spectroscopy results, which also revealed the most pronounced α-helical stabilization in (s)SpA h1. These outcomes are consistent with our hypothesis that lactam-based stapling would reinforce α-helicity and enhance binding affinity to the Fc region.

**Figure 4:**
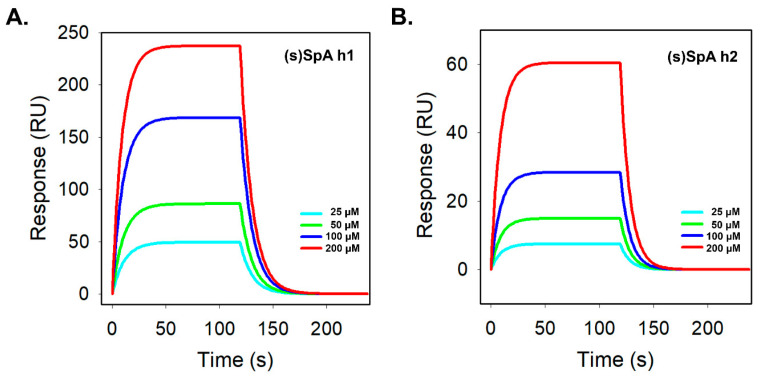
Surface plasmon resonance (SPR) of stapled Fc-binding peptides. SPR sensorgrams depicting the binding interactions of (**A**) (s)SpA h1 and (**B**) (s)SpA h2 with immobilized human IgG in DPBS. Peptides were injected at concentrations ranging from 25 to 200 μM.

To quantitatively evaluate the intrinsic Fc-binding performance of the peptides, surface plasmon resonance (SPR) analysis was conducted. SpA h1 exhibited consistently higher response units (RU) than SpA h2 across all concentrations tested, indicating a fundamentally stronger interaction with the Fc region (Figure S3a). After lactam stapling, (s)SpA h1 did not show a substantial increase in overall binding signal; however, it retained a steep association phase and a slow dissociation phase, reflecting its inherently high affinity toward the Fc region (Figure 4A). In contrast, SpA h2 showed weak binding responses with slow association kinetics, and although lactam stapling improved its helical stability, the stapled variant (s)SpA h2 exhibited only modest binding, reaching ~60 RU even at the highest concentration tested (Figure 4B). Both peptide pairs displayed clear concentration-dependent binding profiles, confirming their specific interactions with the Fc region. Kinetic analysis further demonstrated that SpA h1 already possesses substantial intrinsic Fc-binding capability, whereas SpA h2 exhibits very weak affinity due to an unfavorable interaction interface (Table S1). Notably, (s)SpA h1 maintained binding kinetics similar to its linear counterpart, suggesting that its Fc-contacting residues are already pre-organized into a near-optimal α-helical conformation, which limits the extent of improvement achievable through stapling. These results align with the CD analysis, which indicates that SpA h1 exhibits higher intrinsic helicity than SpA h2, supporting the notion that structural pre-organization contributes to its stable Fc-binding behavior. Thus, while lactam stapling enhances the structural rigidity of SpA h1, it does not markedly increase its intrinsic Fc-binding affinity as measured by SPR. In contrast, (s)SpA h2 showed a much more pronounced functional improvement upon stapling, including a ~10-fold increase in kon and a two-orders-of-magnitude decrease in KD (Table 1), consistent with previous studies demonstrating that lactam stapling can significantly enhance binding affinity [46,47]. Together, these findings demonstrate that lactam stapling is particularly beneficial for peptide sequences with low intrinsic affinity—such as SpA h2—while sequences that are already structurally optimized for Fc interaction, such as SpA h1, show limited kinetic enhancement. Moreover, the stable binding performance of (s)SpA h1 can be attributed to the presence of five critical Fc-contacting residues and the increased structural rigidity introduced by lactam stapling, which collectively contribute to its high thermal and enzymatic resistance. Overall, these results highlight the capability of lactam stapling to reinforce the structural integrity of single α-helical peptides while preserving, or in some cases enhancing, their Fc-binding functionality.

**Figure 5:**
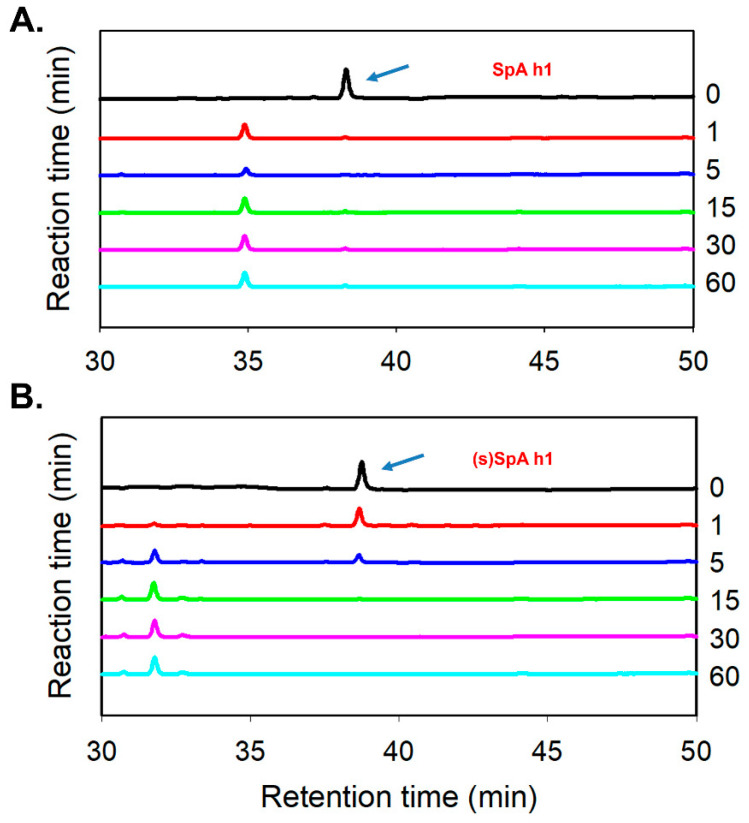
Proteolytic stability of SpA h1 and (s)SpA h1 peptides following α-chymotrypsin treatment. (**A**) HPLC chromatograms of SpA h1 peptide at various reaction times (0, 1, 5, 15, 30, and 60 min). (**B**) HPLC chromatograms of stapled SpA h1 analog, (s)SpA h1, under identical conditions. The arrow indicates the elution peak corresponding to the intact peptide.

Among the synthesized peptides, SpA h1, demonstrating the most pronounced enhancement in both conformational stability and Fc-binding affinity, was subsequently subjected to proteolytic resistance analysis to further evaluate its structural robustness under enzymatic conditions. SpA h1 and its stapled analog (s)SpA h1 were exposed to α-chymotrypsin, and the degradation profiles were monitored by reversed-phase HPLC (Figure 5A,B). SpA h1 underwent rapid degradation, with most of the peptide signal disappearing within just 1 min of enzymatic treatment, indicating high susceptibility to proteolysis. In contrast, (s)SpA h1 retained a considerable portion of its intact peak even after extended incubation, suggesting substantially enhanced resistance to enzymatic cleavage. The enhanced proteolytic stability is attributed to the intramolecular lactam bridge introduced by the stapling strategy, which reinforces the α-helical conformation and renders the peptide less accessible to proteolytic enzymes, thereby not only protecting the peptide backbone from enzymatic degradation but also preserving its functional binding properties [48]. Therefore, the protease-resistant nature of (s)SpA h1 highlights its potential utility as a robust Fc-binding ligand in therapeutic, diagnostic, and antibody purification applications where enzymatic stability is critical.

**Figure 6:**
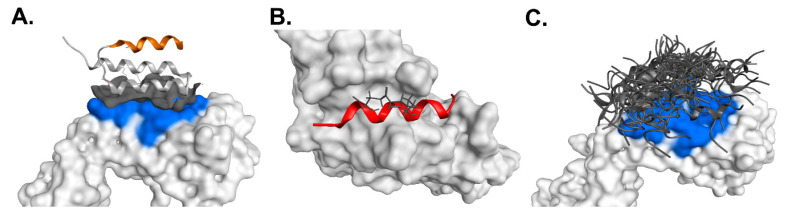
Combined structural modeling and molecular docking demonstrating the Fc-binding specificity of (s)SpA h1. (**A**) Crystal structure of Protein A bound to the Fab region (PDB ID: 1DEE). The orange segment marks the sequence corresponding to the (s)SpA h1 motif, which does not participate in Fab binding. (**B**) Structural modeling of (s)SpA h1 binding to the Fc domain. (**C**) Random placement of (s)SpA h1 on the Fab region for estimating binding affinity. The blue-highlighted area represents the native Protein A binding surface on the Fab domain.

To further elucidate the binding selectivity of (s)SpA h1, we analyzed its structural positioning using the Fab-bound Protein A crystal structure (PDB ID: 1DEE) (Figure 6A). Inspection of this model revealed that the α-helical segment corresponding to the (s)SpA h1 sequence does not make direct contact with the Fab surface, supporting the conclusion that this motif originates exclusively from the Fc-binding interface of Protein A. This structural evidence suggests that (s)SpA h1 is inherently more selective for the Fc region than full-length Protein A. To complement this structural assessment, we conducted MOE-based molecular docking using the Protein A/Fc complex (PDB ID: 1L6X) and compared the binding energetics of (s)SpA h1 toward both the Fc and Fab regions (Figure 6B,C). In this analysis, (s)SpA h1 was not placed randomly; instead, the α-helical segment corresponding to the SpA h1 motif was isolated from the native Fc–Protein A complex and rebuilt based on the same sequence for docking. The resulting Gibbs free energy (ΔG) for Fc binding was –8.34 kcal/mol, which was substantially lower than the ΔG values obtained from multiple docking attempts against the Fab region, as summarized in Table S2. Because (s)SpA h1 does not naturally form a Fab-bound structure, we evaluated all feasible interaction orientations computationally, yet all ΔG values for Fab docking were markedly less favorable than those for the Fc region. Given that lower ΔG corresponds to stronger binding affinity, these results clearly indicate that (s)SpA h1 preferentially recognizes the Fc region over Fab. Collectively, the structural mapping and in silico affinity analyses demonstrate that (s)SpA h1 forms a stable and energetically favorable interaction specifically with the Fc domain, while showing negligible compatibility with the Fab region, including VH3-type domains. These findings strongly support the Fc-selective binding properties of (s)SpA h1 and further underscore its potential as a compact and highly specific Fc-binding ligand.

## 4. Conclusions

In this study, we engineered α-helical Fc-binding peptides derived from the Z34C domain of Staphylococcal Protein A to enhance their structural stability and Fc-binding affinity. To achieve this, a lactam-based stapling strategy was applied to induce intramolecular side-chain cyclization between lysine and glutamic acid residues at positions *i* and *i* + 4, thereby reinforcing the α-helical conformation while preserving key Fc-interacting residues. Circular dichroism (CD) spectroscopy revealed that the stapled peptides, (s)SpA h1 and (s)SpA h2, exhibited significantly increased helical content compared to their unstapled counterparts, indicating successful secondary structure stabilization in solution. Functional evaluation through the IgG capture assay confirmed that lactam stapling enhances Fc-binding performance, with (s)SpA h1 exhibiting the strongest fluorescence signal among all peptide variants. Quantitative SPR analysis further demonstrated that (s)SpA h1 maintains robust and stable Fc interactions consistent with its pre-organized α-helical structure, while (s)SpA h2 benefited more substantially from stapling due to its originally weak intrinsic affinity. Together with the proteolytic resistance assays—where (s)SpA h1 showed markedly improved stability compared to its linear form—these findings validate the successful development of structurally reinforced Fc-binding peptides. Furthermore, our structural analysis and molecular docking results demonstrate that (s)SpA h1 selectively binds the Fc region while showing negligible affinity for the Fab domain, including VH3-type fragments. These findings confirm that (s)SpA h1 functions as a compact and Fc-specific ligand with minimal risk of off-target Fab interactions. Among them, (s)SpA h1 represents the most promising ligand, combining compact size, high structural stability, and selective Fc affinity, making it well suited for applications such as antibody purification, Fc-specific surface immobilization, and site-specific bioconjugation.

## Figures and Tables

**Table 1:** Kinetic binding parameters (kon, koff, KD) derived from SPR measurements.

Sample	kon (1/Ms)	Koff (1/s)	KD (M)
(s)SpA h1	1.02 × 10^2^	8.98 × 10^−2^	8.84 × 10^−4^
(s)SpA h2	2.11 × 10^1^	1.15 × 10^−1^	5.43 × 10^−3^

## Data Availability

All data supporting the findings of this study are available from the corresponding authors upon request.

## Supplementary Materials

The following supporting information can be downloaded at: https://www.mdpi.com/article/10.3390/antib14040108/s1, Figure S1: RP-HPLC chromatograms of the purified peptides. (A) SpA h1, (B) SpA h2, (C) (s)SpA h1, (D) (s)SpA h2. Condition: C18 column, linear gradient from 0 to 50% acetonitrile with 0.1% TFA, flow rate of 2 mL/min, and room temperature. Figure S2: Chemical structure and sequence of (a) SpA h1, (b) SpA h2, and (c) (s)SpA h1, (d) (s)SpA h2. Figure S3: Surface plasmon resonance (SPR) of Fc-binding peptides. SPR sensorgrams depicting the binding interactions of (A) *SpA h1* and (B) *SpA h2* with immobilized human IgG in DPBS. Peptides were injected at concentrations ranging from 25 to 200 μM. Table S1: Kinetic binding parameters (kon, koff, KD) derived from SPR measurements. Table S2: Gibbs free energy (ΔG) values calculated from various docking poses of (s)SpA h1 with the Fab region.
