# Supplementary material for: Enhancement of Structural Stability and IgG Affinity of a Z34C-Derived α-Helical Peptide via Lactam Stapling
Source: Antibodies (Basel). 2025 Dec 16;14(4):108. doi: 10.3390/antib14040108 (PMC12729524; doi:10.3390/antib14040108)
Supplement: Supplementary file 1 [file antibodies-14-00108-s001.zip › antibodies-4009642-supplementary.pdf]

Supporting Information

Enhancement of structural stability and IgG affinity of a *Z34C*-derived α-helical peptide via lactam stapling

Jung Gu Lee[1],†, Inseo Lee[1],†,Woo-jin Jeong[1],[2],*, and Ji-eun Kim[3],*,

[1] Department of Biological Sciences and Bioengineering, Inha University, Incheon, 22212, Republic of Korea

[2] Department of Biological Engineering, Inha University, Incheon, 22212, Republic of Korea

[3] Department of Chemical and Biochemical Engineering, Dongguk University, 30 Pildong-ro 1-gil, Jung-gu, Seoul, 22012, Republic of Korea

†J.G. Lee and I. Lee contributed equally to this work.

Keywords : Lactam stapling, Fc-binding peptide, α-helical peptide, Z34C domain

SpA h1

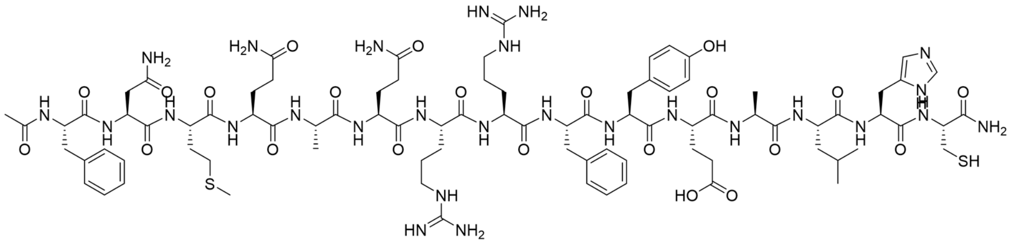

SpA h2

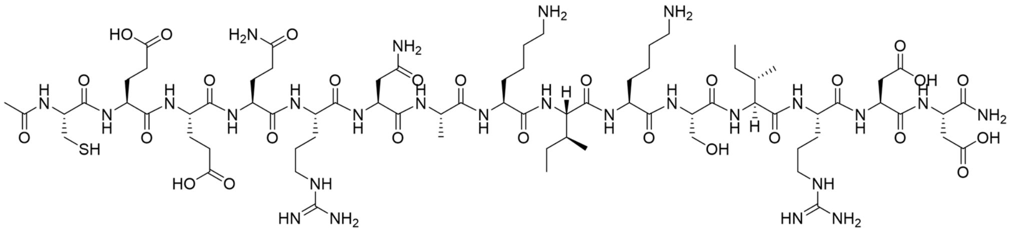

(s)SpA h1

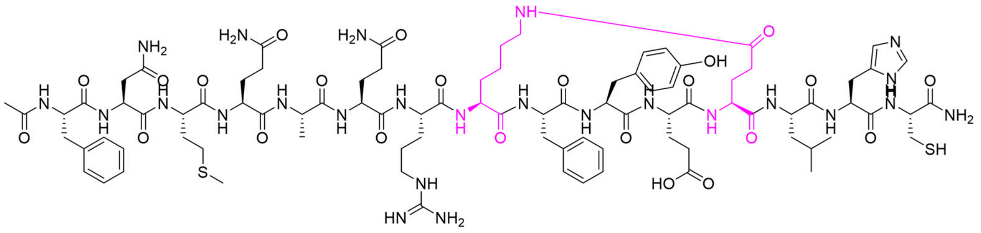

(s)SpA h2

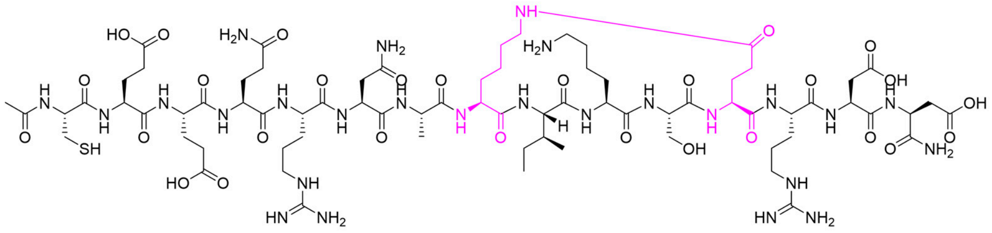

Figure S1. Chemical structure and sequence of (a) SpA h1, (b) SpA h2, and (c) (s)SpA h1, (d) (s)SpA h2.

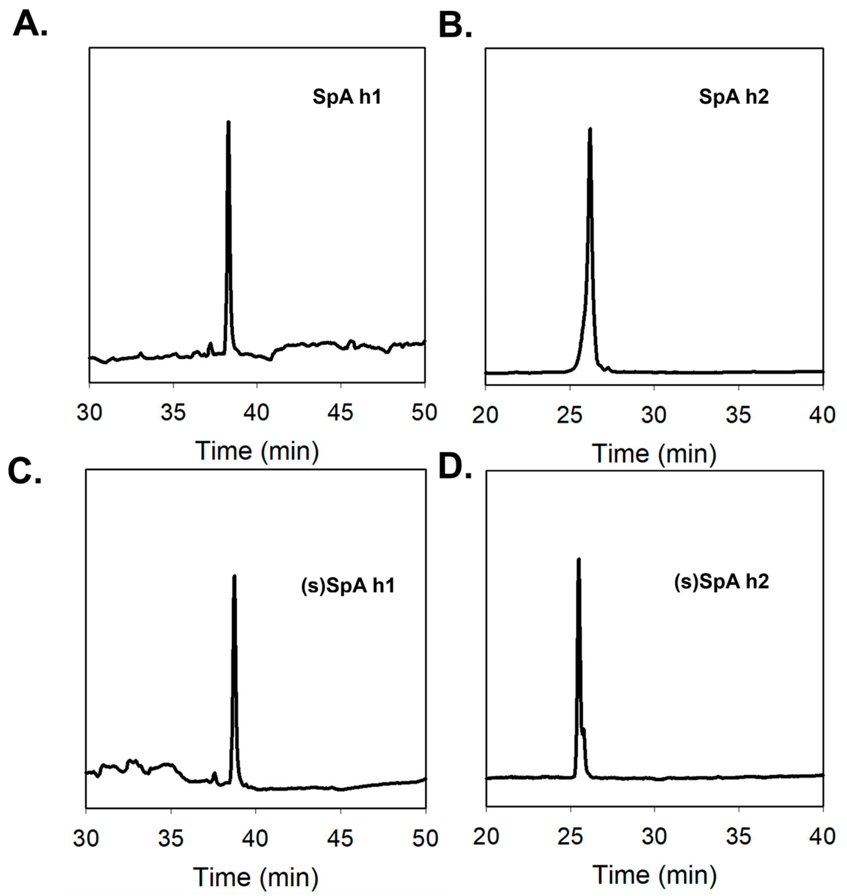

Figure S2. RP-HPLC chromatograms of the purified peptides. (A) SpA h1, (B) SpA h2, (C) (s)SpA h1, (D) (s)SpA h2. Condition: C18 column, linear gradient from 0 - 50% acetonitrile with 0.1% TFA, flow rate of 2 mL/min, and room temperature.

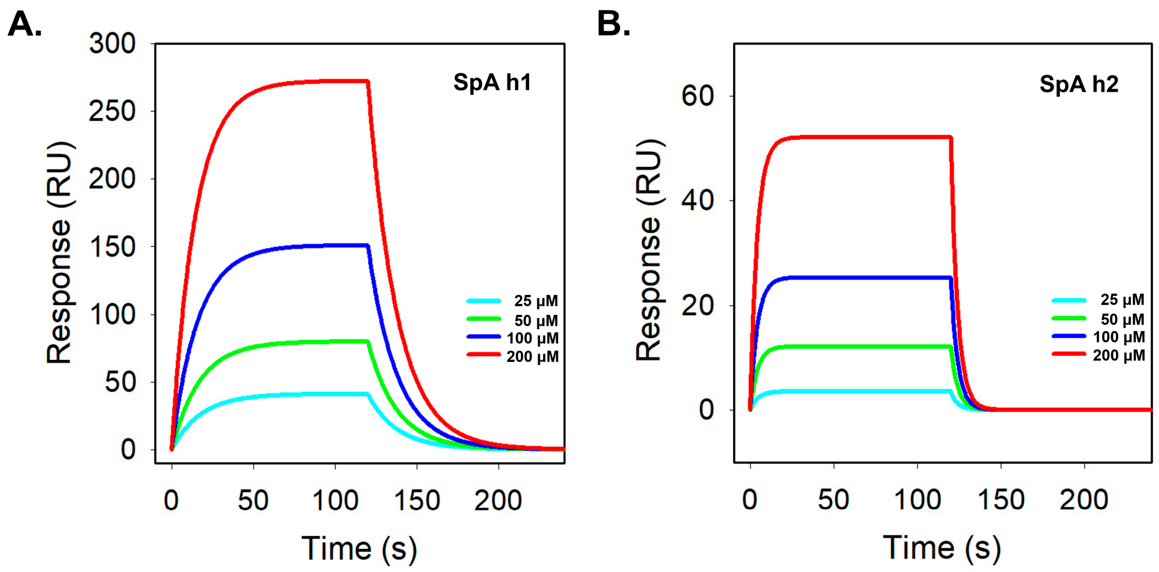

Figure S3. Surface plasmon resonance (SPR) of Fc-binding peptides. SPR sensorgrams depicting the binding interactions of (A) SpA h1 and (B) SpA h2 with immobilized human IgG in DPBS. Peptides were injected at concentrations ranging from 25 to 200 µM.

Table S1. Kinetic binding parameters (kon, koff, KD) derived from SPR measurements.

Sample	kon (1/Ms)	Koff (1/s)	KD (M)
SpA h1	6.82×10^{1}	5.59×10^{-2}	8.20×10^{-4}
SpA h2	2.09	2.40×10^{-1}	1.15×10^{-1}

Table S2. Gibbs free energy (ΔG) values calculated from various docking poses of (s)SpA h1 with the Fab region.

No.	ΔG (kcal/mol)	No.	ΔG (kcal/mol)	No.	ΔG (kcal/mol)	No.	ΔG (kcal/mol)
1	-5.7602	14	-5.5842	27	-5.4311	40	-5.0754
2	-5.7566	15	-5.5840	28	-5.4258	41	-5.0686
3	-5.7216	16	-5.5752	29	-5.4148	42	-5.0407
4	-5.7069	17	-5.5643	30	-5.3621	43	-4.9745
5	-5.7063	18	-5.5571	31	-5.3542	44	-4.9455
6	-5.6722	19	-5.5527	32	-5.3389	45	-4.9094
7	-5.6559	20	-5.5468	33	-5.3386	46	-4.8630
8	-5.6540	21	-5.5086	34	-5.3345	47	-4.7295
9	-5.6452	22	-5.4804	35	-5.3333	48	-4.5916
10	-5.6283	23	-5.4620	36	-5.2971	49	-4.4387
11	-5.6113	24	-5.4586	37	-5.2521	50	-4.0871
12	-5.6003	25	-5.4464	38	-5.1731		
13	-5.5886	26	-5.4428	39	-5.1675		
